# The Role of the Polio Program Infrastructure in Response to Ebola Virus Disease Outbreak in Nigeria 2014

**DOI:** 10.1093/infdis/jiv581

**Published:** 2016-04-02

**Authors:** Rui G. Vaz, Pascal Mkanda, Richard Banda, William Komkech, Olubowale O. Ekundare-Famiyesin, Rosemary Onyibe, Sunday Abidoye, Peter Nsubuga, Sylvester Maleghemi, Bolatito Hannah-Murele, Sisay G. Tegegne

**Affiliations:** 1 World Health Organization, Country Representative Office, Abuja, Nigeria; 2 World Health Organization, Regional Office for Africa, Brazzaville, Congo; 3 Global Public Health Solutions, Atlanta, Georgia

**Keywords:** polio program infrastructure, National emergency operation center, Ebola virus disease, Nigeria

## Abstract

***Background.*** The current West African outbreak of the Ebola virus disease (EVD) began in Guinea in December 2013 and rapidly spread to Liberia and Sierra Leone. On 20 July 2014, a sick individual flew into Lagos, Nigeria, from Monrovia, Liberia, setting off an outbreak in Lagos and later in Port Harcourt city. The government of Nigeria, supported by the World Health Organization and other partners, mounted a response to the outbreak relying on the polio program experiences and infrastructure. On 20 October 2014, the country was declared free of EVD.

***Methods.*** We examined the organization and operations of the response to the 2014 EVD outbreak in Nigeria and how experiences and support from the country's polio program infrastructure accelerated the outbreak response.

***Results.*** The deputy incident manager of the National Polio Emergency Operations Centre was appointed the incident manager of the Ebola Emergency Operations Centre (EEOC), the body that coordinated and directed the response to the EVD outbreak in the country. A total of 892 contacts were followed up, and blood specimens were collected from 61 persons with suspected EVD and tested in designated laboratories. Of these, 19 (31%) were positive for Ebola, and 11 (58%) of the case patients were healthcare workers. The overall case-fatality rate was 40%. EVD sensitization and training were conducted during the outbreak and for 2 months after the outbreak ended. The World Health Organization deployed its surveillance and logistics personnel from non–Ebola-infected states to support response activities in Lagos and Rivers states.

***Conclusions.*** The support from the polio program infrastructure, particularly the coordination mechanism adopted (the EEOC), the availability of skilled personnel in the polio program, and lessons learned from managing the polio eradication program greatly contributed to the speedy containment of the 2014 EVD outbreak in Nigeria.

Ebola virus disease (EVD) is a highly contagious acute viral infection with high mortality rates [1–4], frequently transmitted through close contact with blood, secretions, organs, or other body fluids of infected persons, fomites, and contact with the body of the deceased person [4–6]. Contact with infected semen up to 7 weeks after clinical recovery may also be a source of transmission [7, 8].

The EVD outbreak that began in 2013 and is still ongoing remains the most severe communicable disease outbreak in West Africa in recent times, presenting a challenge to public health systems both within and outside the subregion [9]. The outbreak, which began in Guinea in December 2013, rapidly spread to neighboring Liberia and Sierra Leone [10–13]. Nigeria reported its first EVD case on 20 July 2014, when a sick 40-year-old man flew into Lagos, the main commercial city in Nigeria, from Monrovia, Liberia [14]. On arrival at the Lagos airport, he collapsed and was taken to a private hospital, where EVD was diagnosed on 23 July 2014. The virus then spread to Port Harcourt city in Rivers State, when on 1 August 2014, a symptomatic male contact of the index case secretly flew into the city, igniting another wave of transmission. On 20 October 2014, the World Health Organization (WHO) declared Nigeria free of EVD, 42 days after the last case patient was discharged from the treatment center in Port Harcourt. Nigeria thus had an EVD outbreak for 93 days [15]. By August 2015, other countries that had reported cases from this outbreak were Senegal, Mali, Spain, the United Kingdom, and the United States [16].

The Nigeria's polio program infrastructure consists of partnership between the government and development partners with a reporting system established from the community, local government area (district), state, and federal levels. The structure, originally designed to achieve the Polio Eradication Initiative (PEI) goals through the traditional PEI strategies of a strong surveillance system for acute flaccid paralysis (AFP), high routine immunization coverage with oral polio vaccine, and supplemental polio immunization campaigns, has evolved to incorporate new areas, such as demand creation, operational research, and environmental surveillance [17, 18].

PEI activities in Nigeria are coordinated by the National Polio Emergency Operations Center (EOC), established on 23 October 2012 by the Presidential Task Force on Polio [17–19]. The EOC is a flexible management arrangement that allows for all government agencies, international agencies, local nongovernmental organizations, and the private sector to work harmoniously together in polio eradication. This article describes the response to the 2014 EVD outbreak in Nigeria and how the polio program infrastructure was used to support outbreak response activities.

## METHODS

The major components of the polio program infrastructure used for the EVD outbreak response were the coordination mechanism adopted from the national polio EOC, personnel in the polio surveillance network, and previous lessons learned. The national polio EOC is government led, and its membership includes key officers from government departments and international partner agencies, working on strategy, situational awareness, operations and communication [19].

### Initial Response

At the onset of the outbreak in Lagos State, key officers from the national polio EOC were deployed to Lagos on 23 July 2014 to start the Ebola Emergency Operations Center (EEOC) [15].

### EEOC Organization

The polio EOC deputy incident manager (IM), whose responsibilities were to provide overall coordination of the outbreak response headed the EEOC as the IM for that response and was also the spokesperson for the EEOC, responsible for communication with the media. The EEOC was organized around 6 functional units: management/coordination, epidemiology and surveillance, case management, laboratory services, social mobilization, and points of entry and exit (POE). The IM designated individuals to lead each of these units. Terms of reference were developed, and standard operating procedures were adapted from existing WHO documents [20]. Where these were nonexistent, they were promptly developed.

Training for personnel on EVD outbreak and response was rapidly conducted for individuals in all the units of the EEOC to build capacity and ensure successful containment of the outbreak. Technical officers from WHO and other partner agencies, who had been working together in the polio program, provided technical expertise in training on implementation of the various standard operating procedures for contact tracing, case management, infection prevention and control, safe and dignified burial, decontamination, laboratory services, and POE.

Teams from the EEOC units also trained clinicians, port health officials at POE, airline staff, ship crews, military and paramilitary personnel, and other relevant groups on infection prevention and control, decontamination, contact tracing, and other relevant skills. The government, with support from WHO later expanded training to key healthcare workers from all other states in the country that had no EVD cases.

Each unit had its daily review meeting, and the decisions reached were then shared at the daily evening review meeting of the EEOC. At the end of these meetings, action trackers with designated responsible individuals and timelines were developed. The status of implementation of agreed activity plans were evaluated at subsequent meetings of the EEOC. Each day, the EEOC also prepared a situation report on the outbreak, and the IM shared it with all partners.

### Management/Coordination Unit

In Port Harcourt, the management/coordination unit encouraged the state government to establish an EVD treatment center with laboratory support from the University of Port Harcourt Teaching Hospital and to provide adequate funding and logistics support. The unit reviewed and approved the work plans developed by all other units and coordinated the engagement of private sector companies, particularly from the pharmaceutical and the oil and gas sectors, and private individuals in the EVD outbreak response.

### Social Mobilization Unit

The social mobilization unit's mandate was creating awareness, with a focus on community members around the homes of EVD case patients, contacts, and the most at-risk groups (ie, healthcare providers, morticians, and patients' attendants) and advocacy to key civic, political and religious leaders, heads of media houses, schools, and spiritual healing homes. The unit also trained community informants, school teachers, and healthcare workers on key EVD awareness and prevention practices. Information education and communication materials, such as hand bills, posters, and banners, were produced and distributed, and other health promotion activities included motorized rallies, community dialogue, focus group discussion, health education talks and jingles on radio and television, and health talks in schools, churches, and communities. An Ebola alert call center with toll-free lines was created. Together with the social media, these were used to quell rumors and disseminate accurate information to the populace. WHO supported the production of 2 animated videos in Pidgin and English for use in social mobilization.

### Epidemiology and Surveillance Unit

The epidemiology and surveillance unit was responsible for identification, detailed investigation, and follow-up of EVD contacts using a standard protocol as well as receipt and documentation of EVD rumors and alerts. Other activities included active case searches in health facilities and communities and operational research.

Real-time collection, collation, analysis, reporting, and archiving of contact-tracing data with mobile phones was introduced and was improved continuously based on feedback from field users. The contact database was updated as more contacts were identified and registered. This process was based on the experience of the real-time tracking of polio vaccination teams in northern Nigeria using Android phones. The software used was the open data kit, which has a user-friendly Web interface for form development and offers good technical support [21].

This unit provided daily updates of the numbers of cases, deaths, and contacts followed up. The bulk of the technical personnel in this unit were polio surveillance personnel (ie, community informants, health facility surveillance focal persons, disease surveillance and notification officers, WHO officers, and residents of the Nigeria Field Epidemiology and Laboratory Training Program).

Enhanced surveillance for EVD continued in Lagos and Rivers, the only EVD-affected states, after the end of the outbreak was announced. The unit developed a plan and trained surveillance personnel at all levels on early warning and alert activities. This ensured that EVD surveillance dovetailed into the Integrated Disease Surveillance and Response, a national strategy supported by WHO.

### POE Unit

The POE unit screened travelers for EVD signs at the seaports, airports, and selected international land borders using ThermoScan thermometers (infrared thermometers that measure temperature without contact manufactured by Braun) and a checklist. When the outbreak began, the 2005 International Health Regulations core capacity requirements at POEs in the country had not yet been achieved, and there was no designated POE focal person. These core capacities are obligations concerning designated sea and airports, in relation to routine prevention, control, and response measures to events that may constitute a public health emergency of international concern. WHO staff and other partners worked with the port health unit of the Federal Ministry of Health, supported its activities, in line with the 2005 International Health Regulations, and used experiences from other countries to develop standard operating procedures for screening travelers and case containment and to develop EVD travel advisories, thereby strengthening the Port Health Service and improving emergency preparedness at the POE.

### Case Management/Infection Prevention and Control

The case management unit investigated EVD rumors and alerts, evacuated suspected case patients to the treatment centers, decontaminated homes and buildings visited by suspected and confirmed EVD case patients, collected specimens, requested laboratory tests, and managed confirmed cases, whereas individuals who tested EVD negative in the laboratory were discharged. The unit was also in charge of safe and dignified burials.

### Laboratory Services

Personnel in the laboratory worked in very close collaboration with the case management unit to test all suspected case patients for EVD and other health conditions that may present with similar symptoms and signs. All patients with confirmed EVD whose condition improved with clinical management and who recovered from their illness were again subjected to 2 consecutive laboratory tests for EVD, conducted ≥48 hours apart and were discharged from the treatment facility only when both test results were negative.

### After the EVD Outbreak

After the country was declared Ebola free, training was done for all state epidemiologists, directors of public health, and laboratory scientists in all 34 states not affected by EVD, including the Federal Capital Territory. All states were expected to develop an EVD response plan and also to identify and set up EVD treatment centers.

## RESULTS

A daily review meeting and a daily situation report were used by EEOC for coordination, decision making and information dissemination to stakeholders. An example of the situation report is shown in Table 1. A total of 89 situation reports were produced in the course of the EVD outbreak response. By the end of the outbreak on 20 October 2014, a total of 892 contacts had been followed up, and only 1 was lost to follow-up. Blood specimens were collected from 61 persons with suspected EVD and tested in the designated laboratories; of these, 19 (31%) were positive for EVD, and 58% of the case patients were healthcare workers. The overall case-fatality rate was 40%.

**Table 1.** Daily Situation Report at the Nigeria EEOC, July–October 2014

Reported Data	National	Lagos State	Rivers State
Cumulative cases			
Confirmed	19	15	4
Probable	1	1	0
Suspected	0	0	0
Cumulative deaths			
Confirmed	7	5	2
Probable	1	1	0
Suspected	0	0	0
Case fatality rate (confirmed and probable), %	40	37.5	50
Cumulative confirmed discharges	12	11	1
HCWs			
Cumulative cases in HCWs	11	9	2
Cumulative deaths in HCWs	5	4	1
Contact tracing			
Cumulative contacts listed	892	362	530
Contacts under follow-up	0	0	0
Contact complete (21-d follow-up)	891	362	529
Contacts lost to follow-up	0	0	1
Alerts and rumors			
Reports by HCWs	0	0	0
Reports by others	0	0	0
Investigated with action taken	0	0	0
Laboratory specimen collected by 20 October 2014			
Pending	0	0	0
Tested	61^a^	…	…

Abbreviations: EEOC, Ebola Emergency Operations Centre; HCWs, healthcare workers.

^a^ Testing done at national level only.

**Figure 1.**
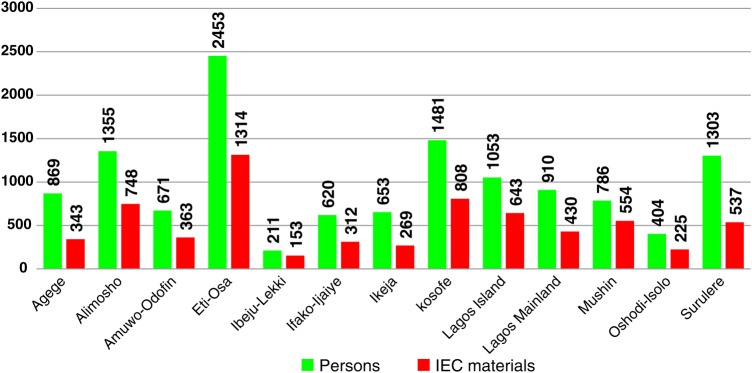
Number of Ebola virus disease information education and communication (IEC) materials distributed and individuals sensitized through person-to-person health education in Lagos from 15 to 23 October 2014.

EVD sensitization and trainings were conducted in 15 of the 20 local government areas in Lagos State, and in all 23 local government areas in Rivers State throughout the outbreak period and for 2 months after the outbreak ended. Figure 1 shows an example of a 9-day communication materials distribution and person-to-person EVD sensitization that was conducted in 15 of the 20 local government areas in Lagos State.

WHO staff from other non EVD-infected states supported the response in the 2 affected states; a total of 25 staff members from other states that work on polio program were deployed in the 2 states for EVD outbreak control. All 5 WHO polio program staff members in Lagos State and 4 of 8 in Rivers State supported the EVD outbreak response.

Each unit of the EEOC responded in an organized manner and rendered daily reports to the strategic group headed by the IM. This allowed for formulation of one mitigation plan with timely allocation of resources, which was designed based on experiences from polio work. Table 2 shows a summary of EVD outbreak response activities conducted by the 6 units of the EEOC.

**Table 2.** Summary of Activities Conducted by the 6 Units of the EEOC for EVD Outbreak Response, July–October 2014 in Nigeria^a^

Sub-unit	EEOC Coordination and Partnership	Epidemiology/Surveillance Unit	Training of Groups	Social Mobilization			Sensitization Meetings	Laboratory Support	POE	Logistics/ Materials and Supplies	Cross Cutting Activities
Activities carried out	Evening review meetings and review of action points using action tracker	Monitoring of disease progression for informed disease	WHO officers	Advocacy visits conducted	IEC materials produced	Video clips	Government and policy makers	Collaboration with laboratory in Lagos	Active surveillance to contain the spread in the airport, seaport, and land crossing	Procurement of PPE	Data collection, collation, analysis, and feedback
	Development of situation report	Daily data collection, collation, and analysis	Other partners and NGOs	Motorized rallies	Banners	Radio jingles	Communities	Use of mobile laboratory to support field work	Advocacy and social mobilization	Procurement of body bags	Trainings
	Information dissemination	Contact tracers	Contact tracers and volunteers	Health education meetings	Flyers	Television shows and education	NURTW and NGOs	Specimen transportation to the laboratory (in Nigeria and abroad)	Screening of pilgrims to Mecca	Provision of adequate transport	Advocacy visits
	Development of standard operating procedures, guidelines, and protocols	Case management of suspected and confirmed cases	Federal/state/LGA/HF surveillance officers	Operational research	Daily review of newspaper	Use of social media (eg, Twitter, Facebook)	Health education in schools (teachers, students, pupils)	…	…	…	Partnership

Abbreviations: EEOC, Ebola Emergency Operations Centre; EVD, Ebola virus disease; HF, health facility; IEC, information education and communication; LGA, local government area; NGOs, nongovernmental organizations; NURTW, National Union of Road Transport Workers; POE, points of entry and exit; PPE, personal protective equipment; WHO, World Health Organization.

^a^ Activities of various subunits of the polio structure repurposed for the 2014 EVD outbreak response in Nigeria.

**Figure 2.**
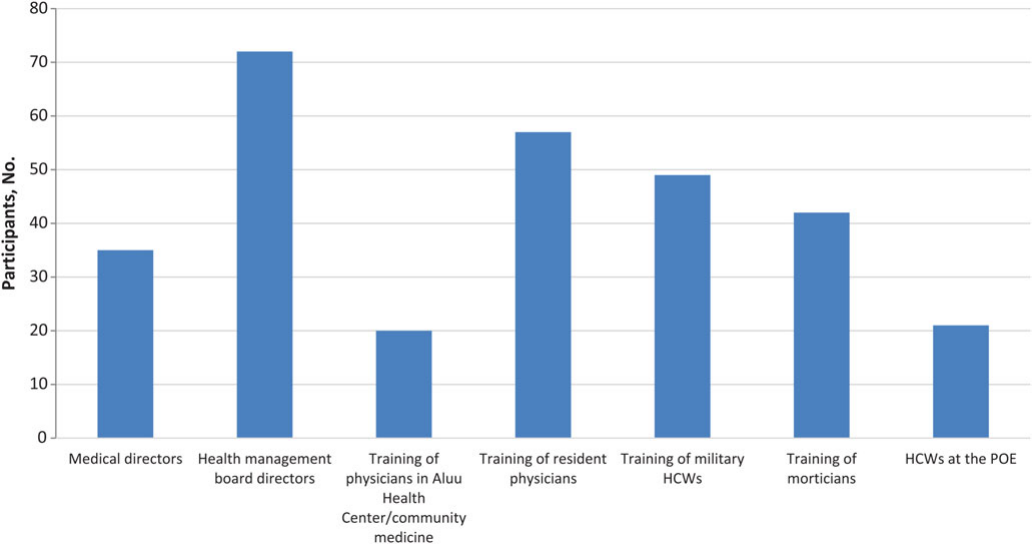
Training of morticians was conducted in two different venues with 22 and 20 participants respectively. Total trained were 42 and can be merged into one. Abbreviations: HCWs, healthcare workers; POE, points of entry and exit.

Different categories of professionals were trained on EVD, including resident physicians, morticians, military personnel, teachers, port workers, government officials, and laboratory scientists. Between the months of September and October 2014, as shown in Figure 2, a total of 296 persons were trained in Rivers State. Various partners contributed vehicles used for the EVD response. Logisticians from the polio program were drafted to manage vehicle movements, fueling, and other supplies under the direct supervision of the EEOC IM, as shown in Table 3.

**Table 3.** Logistical Deployment in Rivers State in September 2014, With Contributions From Different Partners^a^

Supplies and Sources	Quantity Available, No.
Buses (RVSG [hired])	8
4×4 Pickup trucks (RVSG [hired]) and FMOH (n = 4)	16
Ambulances (RVSG)	6
Buses (RVSG) (crew change + 3)^b^	5
WHO (Hired Vehicles)	9
Partners: WHO (n = 4), NFELTP (n = 3), UNICEF (n = 3), Shell Petroleum Development Cooperation (n = 2), Total SA (n = 2), Nigeria Liquefied and Natural Gases (n = 2), and Exxon (n = 2)^c^	18
Jeeps (Exxon Mobil)	2
EEOC Lagos	2
Total	66

Abbreviations: EEOC, Ebola Emergency Operations Centre; FMOH, Federal Ministry of Health; NFELTP, Nigerian Field Epidemiology and Laboratory Training Program; RVSG, Rivers State Government; UNICEF, United Nations Children's Fund; WHO, World Health Organization.

^a^ Status as of 20 September 2014.

^b^ Crews operating the vehicles provided by Rivers State Government and changing shifts.

^c^ These are all direct company/organization vehicles deployed.

## DISCUSSION

Control of the EVD outbreak within 93 days would have been difficult without the effective coordination mechanism adopted - the EEOC, the availability of skilled personnel in the polio program, and lessons learned in managing the polio eradication program, such as how to address noncompliance to polio vaccination, concealing of information, and tracking of suspected AFP cases using mobile device technology. Support and experiences from the polio program structure made the EVD response focused and hence enabled the rapid outbreak containment.

The strong partnership between government and development partners and other nongovernmental organizations, which already existed in the polio program at the EOC, was harnessed to ensure seamless collaboration and expertise in mobilizing the much-needed finances for the EVD response. Informed decisions were quickly reached by the EEOC from data and information gathered using the mobile data devices. These decisions were disseminated equally quickly to stakeholders for prompt implementation.

Lessons learned by the social mobilization unit of the polio EOC, from tackling polio vaccination noncompliance in communities, were leveraged in immediately engaging communities from planning to implementation phase of the EVD outbreak response, production of information education and communication materials, banners, jingles, and other social media. A single source of information dissemination to the general public, a lesson from experiences gained from the polio immunization campaigns, was key in ensuring proper information management and reducing undue panic.

Skilled personnel from the polio program were repurposed to respond to EVD outbreak. They conducted different activities, such as training, contact tracing, social mobilization, operational research, and logistics management, ensuring smooth operations in the EVD outbreak response.

The polio infrastructure has an extensive network of personnel from community to state levels conducting active case searches for AFP. This skill was brought to play in identifying, line listing, and following up of contacts. Interpersonal communication skills often used in obtaining samples for laboratory analysis from difficult AFP cases were also useful in persuading difficult EVD contacts to comply. Data management with real-time mobile technology, which was already being used in the polio program, was also used to ensure timely and complete follow-up of contacts and prompt response to suspected EVD cases.

Replicating our experience in controlling EVD in Nigeria in a short period will require some of the same circumstances that Nigeria had in addition to the WHO infrastructure, which may be a challenge. For example the operating cost of the EVD response, which included payment of personnel, purchase and fueling of vehicles, cost of mobile devices and data bundles, and the daily running cost of the EEOC was prohibitive, which may make it difficult to replicate in other situations without ready resources. Furthermore, setting up the treatment center, aside from being expensive, also caused some communal revolts, because no community in Rivers State wanted the Ebola treatment center in its domain; without proper dialogue, this step could cause a lot of resentment.

We have described how polio resources and experience gained from polio eradication program were used to support the EVD outbreak response in Nigeria in 2014. The PEI program has been instrumental to strengthening the entire health system in Nigeria. In addition, the stronger the health system, the better its ability to respond quickly and mitigate epidemics. The EVD outbreak in Nigeria was rapidly contained because of the leadership from government at the federal and state levels through the coordination of EEOC with full participation of partners to formulate a single mitigation plan and quickly allocate resources. Furthermore, technologies such as geographic information systems assisted the technical officers and resulted in 99.8% success in contact tracing. The polio eradication program made available personnel who were skilled and experienced in surveillance to support multisectoral response under EEOC coordination. We recommend that this strong partnership in EVD response be emulated in tackling future public health emergencies, using resources that already exist in the country. The EOC structure should also be replicated in other states in Nigeria to enable immediate response to any public health emergency.
